# Toward an understanding of occupational burnout among employees with autism – the Job Demands-Resources theory perspective

**DOI:** 10.1007/s12144-023-04428-0

**Published:** 2023-02-25

**Authors:** Michał T. Tomczak, Konrad Kulikowski

**Affiliations:** 1 Faculty of Management and Economics, Gdańsk University of Technology, G. Narutowicza 11/12 Street, 80-233 Gdańsk, Poland; 2 Faculty of Management, University of Social Sciences, H. Sienkiewicza 9 Street; 90-113, Łódź, Poland

**Keywords:** Autism Spectrum Disorders, Neurodiversity, Job Demands–Resources theory, Occupational burnout, Autistic burnout

## Abstract

This article aims to gain insight into the phenomenon of occupational burnout among employees with autism based on the theoretical framework of the Job Demands-Resources theory and the literature on employees with autism in the workplace. Firstly, we argue that although the resources and demands of the neurotypical and neurodivergent employees might be different, the theoretical mechanism of occupational burnout formation remains similar among the neurotypical and neurodivergent employees, leading to the similar burnout experience. Next, we distinguish key demands that might drain neurodiverse employees’ energy, and spark burnout, and propose a set of resources that might foster their achievement of work goals and mitigate demanding working conditions. We emphasise that the nature of job demands/resources that may cause burnout is not universal but might depend on how employees evaluate them, thus neurotypical and neurodiverse workers who evaluate the same work characteristics differently might complement each other, increasing organisational diversity without losing productivity. Our conceptual elaboration contributes to the theory and practice of healthier workplaces by providing tools and inspiration to managers, policymakers, and all stakeholders interested in creating a diverse and productive workplace. Moreover, our work might spark a much needed debate on occupational burnout among employees with autism and encourage conducting further empirical studies.

## Introduction

According to the American Psychiatric Association (2013), Autism Spectrum Disorders (ASD) is characterized by persistent deficits in social communication and social interaction in multiple contexts (such as social-emotional reciprocity; nonverbal communicative behaviors used for social interaction; developing, maintaining, and understanding relationships) accompanied by restricted, repetitive patterns of behavior, interests, or activities. It is worth noting that the above characteristics refer to a very broad spectrum and their occurrence and severity varies greatly from person to person. While individuals with autism are only part of the larger neurodivergent community, which also includes people with ADHD, dyslexia, dyspraxia, Tourette syndrome, and other, for the purpose of this article we use the terms “autism”, “on the autism spectrum” and "neurodiversity" interchangeably and synonymously throughout the manuscript.

Recently, more attention has been devoted to the issues of inclusion of people with autism in the labor market, and numerous studies have emerged pertaining to such problems as e.g. employment trends for individuals with autism (Chen et al., 2015), including employment programs and practices (Farkas et al., 2020; Mpofu et al., 2019), interventions targeted to promote employment (Anderson et al., 2017; Hayward et al., 2019), job retention within this vulnerable group (Brooke et al., 2018), and various workplace accommodations (Tomczak, 2022; Khalifa et al., 2020; Lindsay et al., 2019; Patton, 2019). Furthermore, some articles identified barriers to employment (Mai, 2019), examined the professional experiences of adults with autism (Coleman & Adams, 2018), the use of vocational rehabilitation services (Nye-Lengerman, 2017), social support (Krieger et al., 2012), discussed the perspectives of key stakeholders on employment of individuals with autism (Black et al., 2019), or shared recommendations for employees, employers, policymakers, or employment-related services (Solomon, 2020). Despite this, the academic literature constantly lacks practical and contextualized advice for all stakeholders, both for employees with neurodivergent conditions and for their employers, and the science-practitioner gap is growing (Doyle & McDowall, 2021).

In this conceptual paper, we aim to explore the phenomenon of occupational burnout among employees with autism. Our analyzes not only fit into the above-mentioned broader research gap of employment, labor market inclusion, and well-being of individuals with autism, but also raise an emerging and important issue of burnout among employee on the autism spectrum, a phenomenon which is even more underexplored and surely needs further analyses, which is the original contribution of our paper.

Besides providing an analysis of the resources and demands of employees with autism that might increase their development and health by fostering employability and positive work experience (Burke et al., 2010; Khalema & Shankar, 2014; Saleh & Bruyere, 2018), we also would like to postulate that neurodiverse employees might complement neurotypical employees and we would like to discuss in which situation this complementation might be most successful. Employees with autism might lack some resources and face specific demands, but at the same time they might possess some other resources that are not available for their neurotypical colleagues, e.g., some of employees with autism might have ability to maintain a high concentration for a long time during routine and repetitive tasks, and in this situation, the aspect of work characteristics that is a job demand for the neurotypical employee (e.g. routine task receptiveness) is a job resource for neurodiverse one. This kind of reasoning might help to search for the ideal situation where neurotypical and neurodiverse employees complement each other and workplace neurodiversity becomes an opportunity for improvement rather than a cost for the organisation. In more general terms, our elaboration also suggests that it might be possible to increase organisational diversity without losing productivity.

## Understanding occupational burnout among employees with autism—the JD-R perspective

To better understand occupational burnout among employees with autism, we have adopted a theoretical framework of Job Demands–Resources (JD-R) (Bakker & Demerouti, 2017; Demerouti et al., 2001; Llorens et al., 2006). We suggest that we should not invent new models but think of how to use existing theoretical frameworks and a cumulated body of burnout knowledge to better understand the possible causes and effects of occupational burnout among employees on the autism spectrum. According to the JD-R model, burnout arises as a result of high job demand and low job and personal resources. One of the core ideas of the JD-R that distinguishes it from other job design models (Bakker & Demerouti, 2017) is the assumption that not only some (e.g., control, autonomy, effort, rewards etc.) but all characteristics of work environments might be classified into one of two broad categories i.e. job demand or job resources. Job resources are defined as “*those physical, psychological, social or organizational aspects of the job that may do any of the following: (a) be functional in achieving work goals; (b) reduce job demands and the associated physiological and psychological costs; (c) stimulate personal growth and development*” (Demerouti et al., 2001, p. 501). Whereas job demands “*refer to those physical, social, or organizational aspects of the job that require sustained physical or mental effort and are therefore associated with certain physiological and psychological costs (e.g., exhaustion)”* (Demerouti et al., 2001, p. 501). In addition to job resources and job demand that refers to a work characteristic affecting employees, the JD-R framework also sees employees themselves as active agents within the context of the work environment (Bakker, 2017; Demerouti, 2014). Consequently, JD-R suggests that employee characteristics called personal resources are an important predictor of well-being in the workplace. Research within the scope of JD-R investigating the role of personal resources mainly refers to the aspect of employee personality, e.g., optimism and self-efficacy (Bakker & Demerouti, 2017; Xanthopoulou et al., 2013). However, personal resources might also be perceived as other personal characteristics of an employee that are linked to optimal workplace functioning and resilience as psychological capital, i.e., optimism, efficacy, resilience and hope (Luthans et al., 2006), core self-evaluations i.e. self-esteem, generalised self-efficacy, locus of control, and emotional stability (Judge & Bono, 2001), sense of mastery (Hobfoll et al., 2003) or possibly cognitive abilities (Kulikowski, 2020). Recently, by reviewing the literature on resources, Lee et al., (2020) identified 43 types of personal resources and split them into four subcategories: cognitive personal resources, e.g. knowledge; psychological personal resources e.g. optimism; physical personal resources e.g. personal energy; and career personal resources e.g. seniority. Consequently, personal resources might be viewed as extending beyond psychological personality traits, so, we propose to look at the personal resource in a broad context as similarly to job resources.

**Fig. 1:**
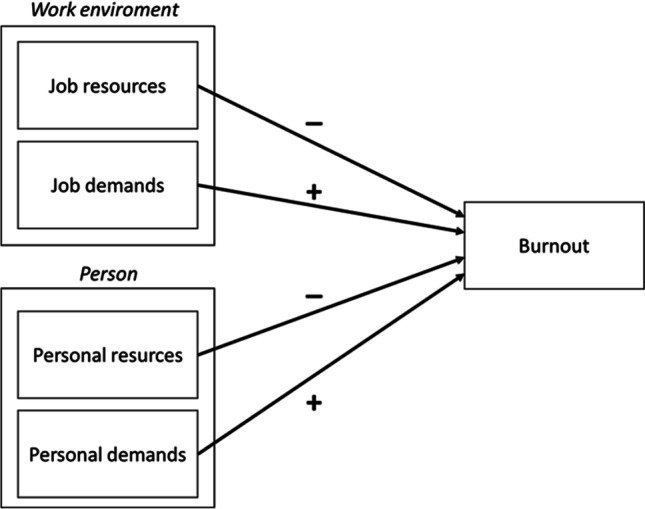
Theoretical model of resources and demands influencing burnout, constituting a framework for our conceptual analysis of burnout among employees with autism based on Job Demands-Resources Theory (Bakker & Demerouti, 2017)

Personal resources are individual characteristics that positively influence employee workplace well-being, but employees might also possess personal characteristics that negatively affect their workplace well-being, i.e. personal demands. The notion of personal demands is currently emerging within research in JD-R theoretical context (Bakker & Demerouti, 2017; Barbier et al., 2013; Salmela-Aro & Upadyaya, 2018). Personal demands similarly to job demands in the JD-R model might not only be psychological but may be defined as all personal characteristics of an employee that generate physical or mental effort and create additional physiological and psychological costs of doing a job (see also Chen & Fellenz, 2020; Zeijen et al., 2021) e.g. high-performance expectation set by employees (Barbier et al., 2013), workaholism (Bakker & Demerouti, 2017), long-term illnesses, caregiving demands, economic problems (Salmela-Aro & Upadyaya, 2018) irrational performance demands, or irrational need for control (Zeijen et al., 2021). Thus, personal demand might be positively associated with burnout. The JD-R model allows us to look at workplace characteristics and, based on these, predict possible causes of burnout among employees with autism by focusing on their special needs and abilities through a theoretical lens of job demands and job resources. But more importantly, the JD-R model allows us to outline individual characteristics that might be most strongly positively and negatively associated with occupational burnout—the individual demands and resources of an employee with autism. Looking at the specificity of employees with autism workplace functioning through the JD-R theoretical lenses might help us untangle the complex relations between workplace characteristics and the formation of their well-being, yielding valuable insights and a better understanding of how to support the development of neurodivergent employees. The conceptual model based on JD-R theory, which integrates job characteristics and personal characteristics of employees with autism in burnout formation is depicted in Fig. 1. This model is based on the JD-R framework and is adapted in such a way as to highlight the most important factors, i.e. autistic job demands, autistic personal demands, autistic job resources, and autistic personal resources that might be related to burnout among individuals with autism. Using this conceptual model, we not only suggest a theoretical framework for autistic employee well-being analysis in organisations aiming at neurodiversity, but building on current literature we also aim to outline those factors that might be particularly strongly related to burnout formation among individuals with autism.

Previously JD-R model was used as an inspiration by Bury et al. (2022) to put forward the Job Demands-Resources for Autism model aiming to understand the relationships between workplace and mental health of autistic employees e.g. co-occurring psychiatric conditions in autism as anxiety, depression and sleep disorders. Here we focused exclusively on occupational burnout among autistic employees, which is not classified as a medical condition (https://www.who.int/news/item/28-05-2019-burn-out-an-occupational-phenomenon-international-classification-of-diseases) and we use JD-R not only to call for adaptation and support of autistic employees (which are important) but also we use JD-R to show possible neuroatypical strength in comparison to the neurotypical workforce. Moreover, Hayward et al. (2020), based on the JD-R theory to better understand autistic social demands and relational resources via qualitative thematic analysis, proved that different facets of social relationships were described as occupational demands by employees with autism compared to neurotypical individuals. Moreover, these demands appear to have a greater impact on the well-being of neurodivergent individuals (Tomczak, 2022).

Consequently, we intend to provide a detailed analysis of possible job demands, job resources, personal demands, and personal resources of employees with autism. This endeavour is important as it might not only provide information for employers and human resources departments on how to support employees with autism but might also help to build a more inclusive workplace creating a situation in which the employment of neurodiverse workers is not only an element of a corporate social responsibly policy but will actually improve the organisation of work. We suggest that taking into account the unique resources and demands of employees with autism might not only help to create a burnout-free work environment, but also positively impact an organisation's long-term performance.

## Burnout vs “autistic burnout” construct clean-up time

Despite the great interest in burnout among researchers and laypeople, it is still not clear how to define burnout and, what is more, there is no single common method for measuring it (see e.g. Halbesleben & Buckley, 2004; Schaufeli et al., 2009a, 2009b; Bianchi et al., 2015; Hakanen & Bakker, 2017; Shoman et al., 2021; Taris, 2006; Taris et al., 2005; Leiter & Maslach, 2016). According to one of the most influential approach, burnout is conceptualised as a response to prolonged job stress characterised by three dimensions, namely exhaustion, cynicism and lack of accomplishment (Maslach et al., 2001). A similar three-dimensional approach is also encompassed by WHO in *International Classification of Diseases* ICD-11 (WHO, 2019), where burnout is defined as “*syndrome conceptualized as resulting from chronic workplace stress that has not been successfully managed*” characterised by energy depletion, mental distance from one’s job and reduced professional efficacy (https://icd.who.int/browse11/l-m/en#/http://id.who.int/icd/entity/129180281). In line with JD-R framework, burnout is developing as a result of the health impairment process, in particular in situations when high job demands consume energy, impede work goals, and generate psychophysiological costs, while job resources are insufficient to meet those demands (see Lee & Ashforth, 1996; Alarcon, 2011; Bakker et al., 2005; Schaufeli & Bakker, 2004; Schaufeli et al., 2009a, 2009b; Bakker et al., 2014; Bakker & Demerouti, 2017).

It is also important to emphasise that by adopting the JD-R approach to understanding burnout among employees with autism, we believe that although the job/personal resources and demands of neurotypical and neurodivergent employees might be different, the mechanism of burnout formation remains simlar, leading to the similar occupational burnout experience. This distinction is important, as lastly, some authors argued about the need of defining a construct they called “autistic burnout” related to life in general, not only to the work context. Raymaker et al., (2020; p. 133), based on qualitative analysis, describe autistic burnout as *“a syndrome conceptualized as resulting from chronic life stress and a mismatch of expectations and abilities without adequate supports. It is characterized by pervasive, long-term (typically 3* + *months) exhaustion, loss of function, and reduced tolerance to stimulus”*. Whereas Higgins et al., 2021, attempted to define “autistic burnout”, suggesting that the two main diagnostic criteria for “autistic burnout” are exhaustion and interpersonal withdrawal with at least one of the following reductions in important areas of functioning, difficulties with cognitive functioning and increased intensity of autistic traits. As two similar constructs “autistic burnout” and “occupational burnout among employees with autism” despite being almost identical in names have different meanings, thus for the sake of clarity, we highlight that in this conceptual analysis, we analyse an occupational burnout among employees with autism—a response of employees with autism to chronic workplace stress resulting from the health impairment process as described in the JD-R model, and that we are not referring here to the emerging concept of “autistic burnout” as exhaustion that stems from a lack of accommodation to the neurotypical environment (see Mantzalas et al., 2022). Consequently, in the following paragraphs, we discuss what aspects of job characteristics constitute job resources and job demands for employees with autism, and by analogy, we analyze what personal characteristics are personal resources and personal demands for representatives of this community.

## What aspects of job characteristics are job resources for an employee with autism?

Schaufeli (2017) proposed the division of job characteristics within the scope of job resources into four main types, such as social resources, work resources, organisational resources, and developmental resources. Within these types, we have indicated those that most accurately reflect the job resources in terms of the specific needs of neurodiverse individuals. First, among social resources, having regard to the important role of the support of co-workers and supervisors in the process of inclusion of neurodiverse people within the work environment (Tomczak et al., 2021), we refer to Schaufeli’s job characteristics (2017) such as *co-worker support, supervisor support*, and additionally, *buddy schemes,* as proposed by Lee et al., (2020). Among the work resources, we focus on the possibility of using the unique abilities of people with autism as a result of different cognitive styles (Tomczak, 2021), including the use of necessary work tools, so as a result, we point to the *use of skills* and *availability of tools* introduced by (Schaufeli, 2017). An important issue in this context is also a clear division of tasks, combined with valuable feedback. This is why we also indicate *goal clarity, role clarity,* and *performance feedback* (Lee et al., 2020). Among the organisational resources, communication issues seem to be of key importance, together with creating a climate of tolerance, ensuring a sense of security, and multi-level support including ​​organisational practices, and also providing the possibility of using own specific skills by each individual (Szulc et al., 2021a; Tomczak, 2021). We, thus, refer to the communication mentioned by Schaufeli (2017), as well as to the following characteristics coined by Lee et al. (2020): *positive workplace climate, psychosocial safety climate, job security, perceived organizational support, supportive professional practice environments, technology support*, and *physical work environmen*t. Finally, concerning developmental resources, we point to *a career perspective,* as suggested by Schaufeli (2017).

## What aspects of job characteristics are job demands for an employee with autism?

When analysing the job characteristics proposed by Schaufeli (2017) concerning job demands that might be most challenging for neurodivergent employees, we focus on the selected qualitative, quantitative, and organisational demands pointed by this author (Schaufeli, 2017). Among the qualitative demands, we highlighted demands associated with a high perceived level of stress (Tomczak et al., 2020) and the difficulties associated with sensory sensitiveness (Tomczak, 2021). Among these demands, we can name *emotional demands, mental demands,* and *physical demands*. Within quantitative demands, we might distinguish characteristics related to over-stimulation and preferences in the area of ​​order and predictability, such as *the pace of change* (Schaufeli, 2017). Finally, concerning organisational demands, we focus on demands resulting from difficulties in the scope of ​​undertaking and maintaining social interactions, thus referring to the demands stated by Schaufeli (2017), e.g. *negative change, harassment,* and *interpersonal conflicts*.

## What personal characteristics are personal resources for an employee with autism?

Some of the personal characteristics of people with autism may be considered as their potential strengths in the context of their work, especially when compared to such characteristics in neurotypical people. First of all, these characteristics relate to their particular interests (Goldfarb et al., 2019), so we can mention *the memory ability* that enables the retention of a large amount of information (CIPD, 2018; Szulc et al., 2021a) and other *highly specialized individual skills,* including reading, drawing, music, computation (Meilleur et al., 2015), *strong detail focus and detail processing* (Dreaver et al., 2020), *systemizing skills and information cataloging* (Baron-Cohen et al., 1999). Neurodiverse individuals may also often have a strong interest in digital technologies (Tomczak et al., 2018), so their personal resources might involve *a technical interest in specific work areas/technical roles*. They also may be capable of *innovative thinking and creativity* due to ability to analyse the problem from a different (non-typical) perspective (Armstrong, 2010), together with *logical/analytical thinking* (Austin, 2018), *visual thinking, and visual skills* (The Institute, 2020) and *detection and reflection toward patterns skills* (Tomczak, 2021). Furthermore, other characteristics may include: *punctuality and reliability* (CIPD, 2018), *maintaining concentration for a long time* (Szulc et al., 2021a), *being focused on long-term recurrent task performing, and tolerance for monotonous actions* (Tomczak, 2021), *honesty* (Szulc et al., 2021a), *loyalty and dedication* (Dreaver et al., 2020). What is more, having in mind sensory hypersensitivity experienced by many people with autism, neurodiverse individuals may be more aware of the needs of others in this area, so their characteristics also include *sensory awareness* (Szulc et al., 2021a).

## What personal characteristics are personal demands for an employee with autism?

Within the personal characteristics that can be perceived as demands for an employee with autism, the issues related to establishing and maintaining interpersonal relationships based on effective communication may be indicated (Tomczak et al., 2021). Therefore, we point to some characteristics identified by previous research, e.g., poor *social skills* (Hayward et al., 2020) and *social reciprocity* (DMA, 2019), difficulties in *interpersonal communication* (Flower et al., 2019), both verbal and non-verbal (DMA, 2019). Some neurodiverse individuals may also face difficulties in *conceptualizing and illustrating abstract ideas* (CIPD, 2018). Other characteristics in terms of ​​demands may be those related to the organisation of work tasks and punctuality, such as *time management* (Wehman et al., 2016), *self-organizing, task prioritizing,* and *multitasking* (Howlin et al., 2005). Another condition arises from anxiety, which is related to operating in unfamiliar situations, and the need for routine (Katz et al., 2015), which may lead to difficulties with flexibility and adapting to changes in structure and routine (Browning et al., 2009). The last possible characteristics address difficulties in susceptibility to stressors, coping with stressful situations, and sensory hypersensitivities. Among them, we indicate *low resistance to stressors and external stimuli* (sound, visual, olfactory, tactile) (DMA, 2019; Szulc et al., 2021b) and poor *stress control skills* (Tomczak et al., 2020). All job and personal characteristics considered as resources and demands of individuals with autism are summarised in Table 1.

**Table 1:** Job resources, personal resources, job demands, and personal demands that may be considered most important for employees with autism

	Example	Source
**Job resources**		
Social resources		
Co-worker support	Co-workers that are prepared to understand the nature of autism and related challenges	Tomczak et al., 2021
Supervisor support	The supervisor who is open for job redesign according to needs of employees with autism	Tomczak et al., 2021
Buddy schemes	On-demand support of one selected co-worker	Tomczak et al., 2021
Work/job-resources		
Use of skills	Skill-based recruitment and selection procedures which ensure that autism-specific skills are matched to job requirements	Tomczak et al., 2021
Availability of tools	Providing the employees with autism with the tools they might need to adapt to the workplace (e.g.headphones to avoid noise, stress monitoring devices)	Tomczak, 2021
Goal clarity*	Precisely defined scope of duties and tasks	Tomczak et al., 2021
Role clarity*	Clear descriptions of work procedures and expected patterns of work behaviours	Tomczak et al., 2021
Performance feedback	Providing direct but sensitive feedback on work performance	Tomczak et al., 2021
Organisational resources		
Communication	Internal communication taking into account the needs of employees with autism (emphasis on non-direct and non-verbal contact)	Tomczak et al., 2021
Positive workplace climate	Supporting an inclusive environment and diversity climate	Tomczak, 2021
Psychosocial safety climate	Organisational and managerial procedures focused on the psychological health and safety of employees with autism	Szulc et al., 2021a
Job security	Providing employees with autism stable job contracts and clearly defined layoff procedures	Szulc et al., 2021a
Perceived organisational support	Acting to ensure all employees with autism that the organisation is dedicated to treating them the same as every other member of the organisation while also being aware of their needs and characteristics	Szulc et al., 2021a
Supportive professional practice environments	Buddy/mentor/job coach assistance (if needed)	Szulc et al., 2021a
Technology support	Non-direct, technology-mediated communication, appropriate adjustments (eg. work in headphones, silent computer keyboards)	Tomczak, 2021
Physical work environment	Providing changes to the work environment to adjust it according to the needs of a person with autism (e.g.chill rooms, avoiding large open spaces and hot desks)	Tomczak, 2021
Developmental resources		
Career perspective	Establishing a clear career path within the organisation for employees with autism, including the positions for which they are predisposed	Szulc et al., 2021a
** Personal resources**		
Cognitive resources		
Memory ability	Retaining a large amount of detailed information	Szulc et al., 2021a
Systemising and cataloguing skills	Ability to organise and catalogue large data sets	Baron-Cohen et al., 1999
Strong detail focus, detail processing*	Attention to detail, accuracy, meticulousness	Dreaver et al., 2020
Innovative thinking and creativity*	Ability to analyse the problem from a different (non-typical) perspective	Armstrong, 2010
Logical/analytical thinking	Strong attachment to logical reasoning in decision-making processes, preference for the slow thinking	Austin, 2018
Visual thinking	Image processing at a high level	The Institute, 2020
High level of specialisation	Narrow and specialised skills in a given field (eg. drawing, music, computation)	Meilleur et al., 2015
Technical interests	High interest in digital technologies	Tomczak et al., 2018
Sensory awareness	Awareness of other people sensory needs (e.g. avoiding noise generation)	Szulc et al., 2021a
Concentration*	The ability to work at a high level of concentration over a long time	Szulc et al., 2021a
Patterns recognition	The ability to faithfully reflect patterns, instructions, and procedures	Tomczak, 2021
Psychological resources		
Honesty*	Not hiding unpleasant facts from someone	Szulc et al., 2021a
Punctuality and reliability	Putting emphasis on punctuality	CIPD, 2018
Being focused on the task	A long-term dedication to a certain activity/not easily bored	Tomczak, 2021
Loyalty and dedication	Ability to maintain long-term cooperation without the need for change	Dreaver et al., 2020
**Job demands**		
Qualitative demands		
Emotional demands	Low level of emotional regulation	Tomczak, 2021
Mental demands*	Difficulties in teamwork	Szulc et al., 2021a
Physical demands	Difficulties in handling external auditory, olfactory and visual stimuli, prone to overload with external stimuli, overstimulation	Tomczak, 2021
Quantitative demands		
The pace of change*	Low tolerance for changes in routine (job duties, work tasks)	Browning et al., 2009
Organisational demands		
Negative change	Changes that need dealing with uncertainty	Katz et al., 2015
Harassment (stigma, bias)	Inappropriate treatment from co-workers because of specific behaviours or needs characteristic for individuals with autism	Krzeminska et al., 2019
Interpersonal conflicts	The co-workers’ lack of awareness of the nature of neurodiversity	Krzeminska et al., 2019
**Personal Demands**		
Cognitive demands		
Problem with conceptualising and illustrating abstract ideas	Struggle to understand metaphors	CIPD, 2018
Low level of time management abilities	Difficulties in the self-organisation of working time	Wehman et al, 2016
Difficulties with multitasking	Difficulties in coping with various tasks at the same time	Howlin, et al., 2005
Non-optimal work task prioritising	Difficulties in setting priorities	Howlin, et al., 2005
Psychological demands		
Self-organising	Difficulties with the self-organisation of work	Howlin, et al., 2005
Flexibility and adapting to changes in structure and routine	Preferring routine, repetitive tasks	Browning et al., 2009
Resistance to stressors and external stimuli (sound, visual, olfactory, tactile)	Easy irritable in response to stimuli	Szulc et al., 2021b;
Stress control skills	Inability to control stress expressions	Tomczak et al., 2021
Social reciprocity	Difficulties with understanding social norms	DMA, 2019
Social skills*	Difficulties in social interactions (eg. participating in small talk)	Hayward et al., 2020
Interpersonal communication	Difficulties in verbal and non-verbal communication that may lead to interpersonal conflict	Flower et al., 2019

* stands for resources and demands of dual nature, which might constitute resources for individuals with autism and at the same time can be perceived as demands for neurotypical individuals, and vice versa

Source: own elaboration based on Szulc et al., 2021a; Szulc et al., 2021b; Tomczak, 2021; Tomczak et al., 2021; Tomczak et al., 2020; Dreaver et al., 2020; Hayward et al., 2020; Lee et al., 2020; The Institute, 2020; DMA, 2019; Flower et al., 2019; Goldfarb et al., 2019; Krzeminska et al., 2019; Meilleur et al., 2015; Austin, 2018; CIPD, 2018; Tomczak et al., 2018; Schaufeli, 2017; Wehman et al., 2016; Katz, et al., 2015; Armstrong, 2010; Browning et al., 2009; Howlin, et al., 2005; Baron-Cohen et al., 1999

## Discussion

Diversity can be an important factor in improving overall group performance due to the increase in creativity and innovation through the team members’ greater variety of perspectives (Tomczak, 2021). On the other side, it can also lead to misunderstandings and conflicts, as heterogeneity in teams can cause a reduction in intra-group cohesiveness (Roberge & van Dick, 2010). Therefore, the ability to use the advantages and benefits of job resources and demands, including the diverse talents and skills of neurodiverse and neurotypical employees is crucial.

### Dual nature of job demands and resources

Both neurodiverse individuals and their neurotypical colleagues have personal characteristics within which we can distinguish the ones that might be perceived vital for the implementation of work tasks and can also promote the occurrence of the phenomenon of occupational burnout. Some of these characteristics are personal resources for neurodivergent individuals and at the same time personal demands for their neurotypical colleagues and vice versa (see Table 1). Accurate selection of tasks that match personal characteristics and preferences can significantly improve work performance. For example, tasks that require intense concentration over a long time, strong detail focus, or that involve repetitive and monotonous activities that may be burdensome for neurotypical individuals, may be interesting and engaging for neurodiverse people, so assigning them such tasks may result in increasing work efficiency. On the contrary, when performing dynamic tasks that require intense interpersonal contact or in an environment exposed to intense auditory stimuli, neurotypical people can successfully support or replace their neurodiverse colleagues, for whom such working conditions are detrimental in terms of work performance. Likewise, precisely defined job responsibilities and clear descriptions of work procedures can increase the sense of security within the work environment and facilitate the day-to-day tasks of employees with autism. At the same time, such solutions may be perceived by neurotypical staff as unnecessary bureaucracy. Going further, tasks that require a non-standard approach and creativity, which in the case of neurodiverse people may result from their ability to analyse a problem from a different (non-typical) perspective, may be another area in which employees with autism can provide great support and complement the work of their neurotypical colleagues. Another example can be a remote form of work that has become widespread in the face of the COVID-19 pandemic. For many neurodiverse people, this form of work may be highly preferred (Mpofu et al., 2022; Szulc, 2022) due to autonomy (Gajendran et al., 2015) and the possibility of reducing external distractors and stressors (Szulc et al., 2021b; Tomczak et al., 2022). In contrast, some neurotypical employees, in addition to the advantages, may also find remote work burdensome due to limited social contact and isolation (Bartel et al., 2012; Ebrahimi et al., 2021), which may not be as severe for some of the people with autism. Similarly, as in the case of remote work, solutions that benefit neurodiverse people may also include independent positions or single work stations without the necessity to work in a team, as the negative consequences of social isolation may be outweighed by the benefits associated with reducing the stress associated with intense interpersonal contact for this group. Finally, sometimes honesty as the ability to present one's opinions objectively and directly, without hiding unpleasant facts, can, depending on the group, be perceived as both a resource and a demand. Dealing with this direct form of communication can be challenging for neurotypical people, but it should be noted that it is natural for some individuals with autism. This, incidentally, can also lead to misunderstandings and even cause neurodivergent individuals to be sometimes perceived as, for example, rude or not very empathetic.

Therefore, it is important to provide tailored sensitivity and awareness training programmes for all stakeholders—neurodiverse employees and their neurotypical colleagues, including managerial staff (Tomczak et al., 2021) to obtain the ability to skilfully use the dual nature of job resources and demands. The awareness of differences and various characteristics is a prerequisite for capacity building and establishing successful collaboration based on mutual understanding of needs as well as using one's strengths and reducing weaknesses. This can serve as the foundation for an improvement in the group performance and business success of the entire organisation, and at the same time may also contribute to counteracting the phenomenon of occupational burnout within these specific groups of employees. Furthermore, results of a recent study by Bury et al. (2022) investigating the complex interaction between mental health and well-being, autism characteristics, and the workplace through the lens of job demand-resource theory clearly indicate the importance of developing specific mental health supports in autism employment programs and initiatives**.**

It is also important to note that employees with autism create a highly heterogenous group in relation to their social functioning, patterns of behaviour, interests, or activities (see ICD-11 autism https://icd.who.int/browse11/l-m/en#/http%3a%2f%2fid.who.int%2ficd%2fentity%2f437815624). To this end JD-R framework with its flexible assumptions in relation to demands and resources is suited very well for dealing with such heterogeneity. However, as we provided examples of various possible job/personal resources and demands for an employee with autism, we do not picture every employee on the autism spectrum as having them all. Based on our conceptual elaboration, the employee with autism should not be stereotyped. We only suggest that according to autism diagnosis criteria and research on autism in the workplace, there is a higher probability of having some specific personal characteristics among employees with autism than among neurotypical employees, but when dealing with individual employee, we always must have a personalized approach to his/her individual demands and resources. Nevertheless, we believe that our conceptual elaboration presented in this paper might increase awareness of managers and HR specialists on employees with autism strengths and challenges, constituting a first step in building better, a more neurodiversity-inclusive workplace.

### Implications and limitations

In this conceptual paper, we aim to get insight into the phenomenon of occupational burnout among employees with autism, drawing from the theoretical framework of JD-R theory and the literature on employees with such characteristics functioning in a workplace. Drawing inspiration from the concepts of demands and resources acting as casual factors in burnout formation, we distinguished the most important personal and job demands that might drain neurodiverse employees' energy and increase the probability of burnout. What is more, we proposed a set of personal and job resources that, in the case of employees with autism, might foster the achievement of work goals and mitigate demanding work conditions, thereby decreasing the probability of burnout development (see Table 1). Our conceptual elaboration presented in this paper contributes to the theory and practice of healthier and more diverse workplaces in several ways.

Our analysis might provide tools and inspiration for managers, policymakers, and all stakeholders interested in increasing diversity, creating a more neurodiverse-friendly workplace, and establishing tailored HR practices to achieve this goal, ensuring appropriate support to overcome workplace social challenges experienced by employees on the autism spectrum (Bury et al., 2020). An earlier JD-R model-based study by Stirpe et al., (2022) has proved that satisfaction with HR practices influences role performance. Thus, we propose a ready-to-use list of potential demands and resources which may be available for employees with autism, i.e., factors that facilitate or hinder the positive work experiences of this specific group. Therefore, our suggestions might act as a checklist and a discussion starting point for managers and HR departments when building diverse workplaces that do not exclude neurodiverse employees. However, we not only provide a list of possible demands and resources, but more importantly suggest that organisations caring about diversity can keep the well-being of neurodiverse employees in mind in terms of JD-R theoretical assumptions. Adopting the JD-R framework provides an opportunity to understand the general principles of burnout formation and apply them in different organisational contexts to improve the work experience of employees with autism.

Our elaboration might also serve didactic purposes and help in the diversity training design process for managers and employees. By rising awareness of the most important job demands and resources, our analysis might also be helpful in job crafting interventions and personal resources interventions, i.e., the actions that employees might take to increase resources and reduce demands that help them to boost their well-being in the workplace (see Tomczak, 2022; Tomczak et al., 2022). Possessing the list of possible job demands and resources, it might be easier for employees to understand what factors might lead to burnout and to make changes that might positively affect their well-being. Moreover, it might also help neurotypical and neurodiverse employees to understand each other due to their awareness that different aspects of the job might be demanding and resourceful for each group.

Moreover, the results of our research might be helpful in the process of improving the methodology of employee opinion surveys in organisations aiming at diversity. In the case of burnout, opinion surveys are often useful for collecting employee suggestions about aspects of the job that are most demanding and stressful; unfortunately, this data is analysed through generalisation, e.g.calculating sums or mean values from the responses of all employees. However, as the same job characteristics might be perceived differently by a neurotypical and neurodiverse employee, we should analyse employee surveys results separately for neurotypical staff and employees with autism. Making generalisations add simplicity but blurt out the opinions of neurodiverse employees that usually are minorities. As both groups of employees might see the same work aspects very differently, thus it might be a good idea to not only establish what characteristics of a job need change but also for whom.

From a theoretical point of view, our elaboration agreed with previous voices that the job and personal characteristics might not be unequivocally positive or negative (see Van den Broeck et al., 2010; Li et al., 2020; Van Veldhoven et al., 2020). Different jobs and organisations might not only create their specific job demands and resources, but in the context of a single position, different employees might experience specific resources and demands under “objectively” the same work conditions. For example, previous studies have shown (Crawford et al., 2010) that the impact of job demands on employees’ well-being depends on employees’ appraisal of stressors. When demands are perceived as hindrances, they drain energy and most often lead to exhaustion, but when they are seen as challenges, they might stimulate work engagement. In a similar vein, we suggest that in the case of neurotypical and neurodiverse employees, common job resources might turn into demands, and job demands into resources. For example, a task routine might be perceived as demanding by the neurotypical employee but as a resource by a neurodiverse individual, or close and intense social interaction at work might be seen as a resource by the neurotypical employee and as a demand by a neurodivergent person. Highlighting the dual nature of job demands and resources might explain why the same job characteristics might be perceived differently by both analysed groups of employees and how neurodiversity might be a factor influencing workplace stressors appraisal. In more general terms, our elaboration also suggests that it might be possible to increase organisational diversity without losing productivity. In the case of increasing diversity in business organisations, sometimes there may appear a “productivity vs diversity” dilemma, where an increase in diversity comes at the expense of productivity, as it requires the organisation to bear additional costs. However, taking into account the dual nature of job demands and resources, one might expect that having a more diverse workforce will decrease the burnout incidence in the organisation due to the increase of the probability that employees will be complementary concerning their job demands appraisal. Increasing diversity might be seen not as a cost but rather as an investment in assigning people with tasks that match their expectations, which protects against losses in productivity caused by lack of work engagement. From this perspective, the diversity and inclusion of employees with autism are not only “charity work” aiming to help neurodiverse employees to adapt to work, but also to help business organisations keep high productivity (Austin & Pisano, 2017).

Our original contribution to the theory is also a call for a critical look at previous studies on “autistic burnout” as non-work exhaustion of people with autism (see Higgins et al., 2021; Raymaker et al., 2020). We suggest that the “autistic burnout” concept is vague, and that moving burnout beyond the workplace could be misleading and could hinder understanding of the experience of employees on the autism spectrum. To this end, our work might also contribute to the literature on management and autism as it calls for discussion and clarification on the concept of “autistic burnout” as the experience of people on the autism spectrum beyond the workplace context. As mentioned earlier, recently, some authors suggest and discuss the construction of “autistic burnout” (Mantzalas et al., 2022; Higgins et al., 2021; Raymaker et al., 2020) as the experience of exhaustion and social withdrawal among the autistic community that is not due to work but due to a lack of awareness and acceptance within society and the lack of fit of people with autism in the neurotypical environment. This form of “autistic burnout” is still ill-defined and is not directly related to the workplace, as people with autism who do not work might declare the experience of this form of “burnout”. The detailed discussion of a new concept of “autistic burnout “ resulting from life in the neurotypical world, exceeds the scope of this paper, but we would like to suggest that in our view moving burnout beyond the context of work could create more heat than light. The concept of “autistic burnout” fits into a trend of inviting different types of “burnouts” outside the workplace e.g. “parental burnouts” (Mikolajczak et al., 2020) or “caregiver burnout” (Gérain & Zech, 2019). These “burnouts” are different constructs that occupational burnout with their causes and consequences and labelled them as instances of “burnout”, might cause more confusion in already messy occupational burnout research. We see the use of the term burnout for non-work-related experiences as unfortunate (but for the opposite view see Bianchi et al., 2014). Burnout is best described as a construct strictly related to the workplace, e.g. WHO in the newly developed International Classification of Diseases, ICD-11 clearly states that: “*Burnout refers specifically to phenomena in the occupational context and should not be applied to describe experiences in other areas of life*” (https://icd.who.int/browse11/l-m/en#/http://id.who.int/icd/entity/129180281). Referring to “burnout” as a trendy term might focus our attention on autistic community challenges, but in the long run, it could diminish our understanding of autistic experiences. For example, using the same label of “burnout” for challenges that stem from the work environment and from society in general, might severely interrupt burnout diagnostics and impede the development of appropriate support interventions. Also, in the face of the proliferation of burnout conceptualisation, e.g., Rotenstein et al., (2018) found more than 140 unique burnout definitions, adding another one in the form of “autistic burnout” is, in our view not going to improve our understanding of occupational burnout among employees with autism. In our view, the ideas hidden under “autistic burnout” to avoid workplace conation and improve its clarity and validity might be better represented by “autistic exhaustion” label. We believe that discussion and clarification are particularly important now, in the early stages of “autistic burnout” research. Thus in contrast to the emerging line of literature on more general life-related “autistic burnout”, we analyzed autistic burnout in its standard meaning (see WHO ICD-11) as an occupational burnout among employees with autism.

Clearly, our conceptual work is also not free from several limitations. First of all, as was mentioned before, we are aware that people on the autism spectrum constitute a highly heterogeneous group, and it is not possible to develop one universal set of characteristics describing each of them. However, as caution should be exercised in generalizing, some shared challenges are common and JD-R model is well suited to dealing with the heterogenity of job/personal demands and resources; thus, our list of potential demands and resources of employees with autism can be a starting point for further analysis. Furthermore, our analysis of the literature did not take the form of a systematic review, but it was due to the fact that, so far, the occupational burnout among employees with autism phenomenon has hardly been undertaken by other scholars as a research topic. It is an emerging issue that is just becoming the subject of researchers' interest. Moreover, we presented a theoretical model based on conceptual reasoning, and although it is built on the solid grounds of JD-R theory this model needs further verification in qualitative and quantitative research among employees with autism, this allows for empirical verification of our propositions and this research should include voices of neurotypical and atypical employees.

## Conclusions

In conclusion, in this manuscript we aim to propose a set of job resources that might help protect employees with autism from burnout; this attempt might help to provide positive work experience for neurodiverse employees and contribute to a better performance of organisations that aim to increase diversity. Our conceptual elaboration contributes to the development of a more diverse organisation as it not only provides new insights but also might spark a debate on burnout among employees with autism and encourage conducting further empirical studies.

## Data Availability

Data sharing not applicable to this article as no datasets were generated or analysed.
